# Pain Management Following Total Hip Arthroplasty With Percutaneous Auricular Stimulation (Neuromodulation): A Randomized, Double-Masked, Sham-Controlled Pilot Study

**DOI:** 10.7759/cureus.78920

**Published:** 2025-02-12

**Authors:** Brian M Ilfeld, Engy T Said, Brenton S Alexander, Scott T Ball, Baharin Abdullah, Evan J Jensen, Adam Schaar, John J Finneran

**Affiliations:** 1 Anesthesiology, University of California San Diego, San Diego, USA; 2 Anesthesiology, Outcomes Research Consortium, Houston, USA; 3 Orthopedic Surgery, University of California San Diego, San Diego, USA; 4 Anesthesiology, Scripps Mercy Hospital San Diego, San Diego, USA

**Keywords:** analgesia, non-opioid analgesic, postoperative pain, post-surgical pain, surgical analgesia

## Abstract

Objectives

Percutaneous auricular neuromodulation involves implanting electrodes around the ear and administering an electric current. A device is currently available in the United States, cleared to treat symptoms from opioid withdrawal, with multiple reports suggesting a possible postoperative analgesic effect. This randomized, controlled pilot study aimed to (1) assess the feasibility of a postoperative auricular neuromodulation protocol and (2) provide an estimate of its treatment effects on postoperative pain and opioid consumption following total hip arthroplasty.

Methods

Adults undergoing unilateral, primary, total hip arthroplasty received an auricular neuromodulation device (NSS-2 Bridge^TM^, Masimo, Irvine, California) applied following surgery. Participants were randomized to five days of either electrical stimulation or sham in a double-masked fashion and discharged home with their devices *in situ*. Participants or their caretakers removed the devices at home.

Results

One participant randomized to active treatment removed the device the morning of postoperative day one and withdrew from the study prior to any data collection. The remaining 29 participants were included in the analysis. For the first primary outcome measure, the median (IQR) pain level in the first five days for those receiving active stimulation (n=14) was 2.5 (1.0, 3.8) versus 3.0 (1.9, 4.0) for the sham group (n=15) (P=0.721). Concurrently, the median oxycodone use for the active stimulation group was 3.5 mg (0.1, 9.5) compared to 9.0 mg (2.0, 15.3) for the sham group (P=0.263). No statistically significant differences between treatments were identified for any of the secondary outcome measures. The protocol was successful regarding participant recruitment, intervention administration, data collection, outcomes assessment, and analysis. Six participants (three from each treatment group) removed their device prior to postoperative day five due to either difficulty sleeping while using the device or pain at one of the electrode sites.

Conclusions

While this randomized, controlled pilot study demonstrated the feasibility of using percutaneous auricular nerve stimulation following total hip arthroplasty for both the inpatient and outpatient portions of the postoperative period, it failed to identify improvements in analgesia, opioid-sparing, or pain interference in psychological and physical functioning. Therefore, it remains unclear whether a definitive clinical trial is warranted to investigate its use following total hip arthroplasty. Further research is advisable, possibly with a different auricular neuromodulation device and larger sample sizes.

## Introduction

Providing analgesia following total hip arthroplasty (replacement) is challenging due to the myriad of peripheral nerves innervating the joint, limiting the practicality of peripheral nerve blocks. A potential alternative for pain relief is percutaneous auricular nerve stimulation (also known as "neuromodulation"), which involves implanting electrodes around the ear and using an external pulse generator to deliver an electrical current [1]. Although the exact mechanism of action is not fully understood and is still being researched, it is believed to modulate several neurotransmitter pathways, leading to the release of substances such as norepinephrine, serotonin, and endogenous opioids like beta-endorphins [2,3]. This form of neuromodulation also has effects on pain perception, as well as anxiety and depression [2].

A percutaneous auricular neuromodulation device is cleared by the United States Food and Drug Administration (US FDA) to alleviate opioid withdrawal symptoms (NSS-2 Bridge^TM^, Masimo, Irvine, California) [4]. This device presents numerous advantages: it is straightforward to use (it requires no patient actions), does not require specialized training or extra equipment, and has few contraindications. Unlike opioids, it avoids systemic side effects and eliminates the risks of overdose, dependency, misuse, or diversion. Additionally, it is more affordable than ultrasound-guided neuromodulation devices and can simultaneously target pain from multiple peripheral nerves with a single device.

Two pilot studies published previously reported improved analgesia after cholecystectomy, hernia repair, and total knee arthroplasty, as well as opioid-sparing after knee arthroplasty [5,6]. In contrast, two other studies focusing on colorectal surgery and cesarean section failed to identify positive results for their primary or most secondary outcomes [7,8]. As a result, the applicability of percutaneous auricular neuromodulation cannot be assumed for all surgical procedures. It might be particularly appropriate for hip arthroplasty, given the limited value of peripheral nerve blocks due to the complex innervation of the hip joint.

We, therefore, conducted a randomized, double-masked, sham-controlled pilot study to investigate the use of percutaneous auricular neuromodulation for pain following total hip arthroplasty. We aimed to better inform the planning of a subsequent definitive trial by (1) determining the feasibility of and optimizing a study protocol and (2) estimating the treatment effect.

## Materials and methods

This study followed Good Clinical Practice and was conducted within the ethical guidelines outlined in the Declaration of Helsinki. The study was prospectively registered at ClinicalTrials.gov (NCT05521516). The protocol was approved by the Institutional Review Board of the University of California San Diego, La Jolla, California, USA (approval number: 802775). The Institutional Board determined that the auricular stimulator is a non-significant risk device per the criteria outlined in 21 CFR 812.3(m) and therefore approved the off-label use of this device to investigate its potential to provide postoperative analgesia. Written informed consent was obtained from all participants.

Enrollment was offered to adult patients at least 18 years of age scheduled for primary, unilateral, total hip arthroplasty. Patients were excluded for (1) chronic opioid use inclusive of tramadol (daily use within the two weeks prior to surgery and duration of use greater than four weeks); (2) neuro-muscular deficit of the ipsilateral lower extremity; (3) history of opioid misuse or dependence; (4) concurrent use of another electric stimulator (e.g., cardiac pacemaker); (5) history of bleeding disorder; (6) anticoagulation condition and/or therapy; (7) skin abnormality at the treatment site; (8) psoriasis vulgaris; (9) incarceration; (10) pregnancy; or (11) inability to contact the investigators during the treatment period, and vice versa (e.g., lack of telephone access).

**Figure 1:**
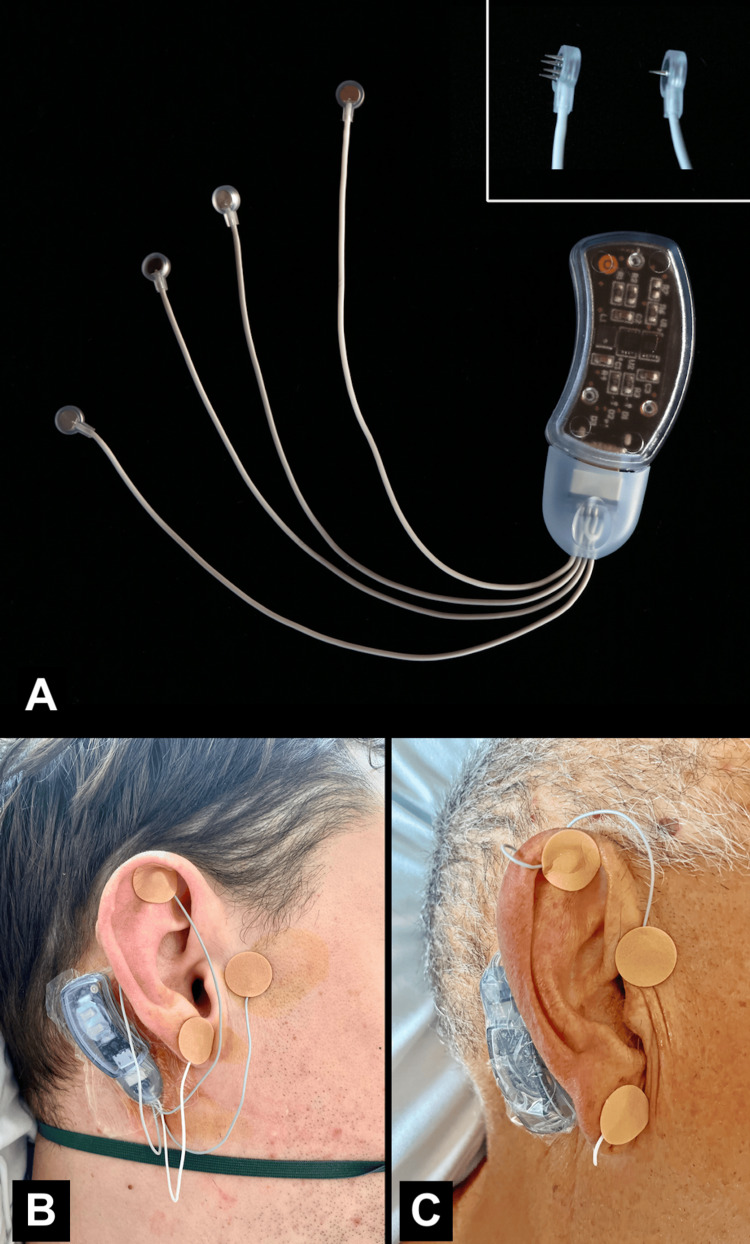
A percutaneous auricular nerve stimulation system (A) Percutaneous auricular nerve stimulation system (NSS-2 Bridge^TM^, Masimo, Irvine, California, USA). Each of the three electrodes has a 2-mm-long integrated needle/lead (inset), and the ground electrode has four 2-mm-long integrated needles/leads (inset). (B, C) Auricular nerve stimulation system during use. Image credits: Brian M. Ilfeld

An investigational pharmacist (University of California San Diego, La Jolla, California, USA) created the randomization list in blocks of two and a 1:1 allocation into the active and sham treatment groups. Active and sham devices (NSS-2 Bridge^TM^, Masimo, Irvine, California, USA) (Figure 1) appear identical and were provided directly to the investigational pharmacist from the manufacturer, differentiated only by serial number. The investigational pharmacist labeled each device with the appropriate randomization number, and no investigator, clinical staff, or participant was aware of the treatment group assignment until study completion.

The participants were randomized postoperatively within the recovery room. The study device was affixed to the ear and activated prior to discharge from the recovery room. There is currently no consensus regarding the placement on the ipsilateral or contralateral ear relative to the surgical procedure. Therefore, the device was placed on the side that the participant sleeps on least. Removable clips were used to hold any protruding hair away from the treatment sites. The external pulse generator was placed posterior or inferior to the ear using benzoin, the included adhesive pad, and an occlusive dressing. The wire harness was inserted into the external pulse generator, which initiated the passage of electrical current (for the participants allocated to the active treatment group). The four electrode locations were cleaned with an alcohol pad, and then a skin protectant wipe was applied (SurePrep, Medline, Northfield, Illinois, USA). A medical light was used to transilluminate the antihelix, and the two electrodes on the cephalad half of the ear were placed 1-3 mm from a neurovascular bundle and never immediately opposite each other.

The first lead was placed at the most cephalad portion of the antihelix by simply pressing the electrode directly into the skin, similar to a thumbtack (Figures 1B, 1C). The second electrode was inserted immediately cephalo-anterior to the incisura and either anterior or posterior to the superficial temporal arterial pulse. The third electrode was inserted on the posterior ear opposite the antihelix at the level of the incisura. The ground electrode with four 2-mm-long integrated needles was inserted on the anterior side of the lobule. No local anesthetic was administered. Benzoin and small round bandages were used to secure the electrodes. If there was discomfort from any of the electrodes, that specific electrode was repositioned.

Postoperatively, the patients received acetaminophen 975 mg three times daily, celecoxib 200 mg twice daily, and, if needed, the synthetic oral opioid oxycodone (5 mg tablets). The patients were instructed to keep the pulse generators and electrodes dry with the use of a shower cap when bathing. Prior to discharge, the participants and their caretakers were provided with verbal and written instructions and the telephone and pager numbers of an investigator available at all times while the device was in use. The participants were discharged home with their electrodes *in situ* and with a prescription for immediate-release oral opioid tablets (oxycodone 5 mg).

The pulse generators automatically ceased functioning after 120 hours (five days). The patients or their caretakers then detached the device by first removing the round bandage of the grounding electrode, which extracted the electrode from the patient along with the bandage. Subsequently, the remaining three electrodes were removed in the same manner, followed by the pulse generator, after which the single-use, disposable device was discarded. Following study completion, the results were provided to all participants using non-technical language.

The participants were contacted by telephone daily for the first eight postoperative days to collect endpoints. We selected outcome measures that have established reliability and validity, with minimal inter-rater discordance, and are recommended for pain-related clinical trials by the World Health Organization and the Initiative on Methods, Measurement, and Pain Assessment in Clinical Trials (IMMPACT) consensus statement [9].

The dual primary outcome measures were the (1) cumulative oral opioid consumption (in oxycodone equivalents) and the (2) mean value of the "average" daily pain scores measured on the 0-10 numeric rating scale (NRS) within the initial five postoperative days. The NRS is a highly sensitive measure of pain intensity with numbers ranging from 0 to 10 (0 = no pain; 10 = worst imaginable pain). It is a valid and reliable measure for evaluating analgesic interventions [10]. Additionally, NRS scores correlate well with other measures of pain intensity [11] and demonstrate high test-retest reliability [12]. These NRS characteristics led to the World Health Organization and IMMPACT consensus recommendations for the use of the 10-point NRS of pain intensity for pain trials [9].

For secondary outcomes, the primary instrument was the Brief Pain Inventory (short form), which assesses pain and its interference with physical and emotional functioning [13]. The instrument includes three domains: (1) pain, with four questions using an NRS to evaluate four pain levels (current, least, worst, and average; collected postoperative days one to eight); (2) percentage of relief provided by pain treatments with one question (not utilized for this study); and (3) interference with physical and emotional functioning using a 0-10 scale (0 = no interference; 10 = complete interference; collected postoperative days two, four, six, and eight). The seven interference questions involve general activity, mood, walking ability, normal work activities (both inside and outside the home), relationships, sleep, and enjoyment of life [13]. These seven functioning questions can be combined to produce an interference subscale (0-70). The use of both single items (e.g., mood) and composite scores is supported by the IMMPACT consensus recommendations for assessing pain in clinical trials [9,14]. Opioid consumption and awakenings due to pain were also recorded during each phone contact.

This investigation was designated *a priori* as a pilot study to assist in planning a subsequent definitive trial. We, therefore, used a convenience sample of 30 participants undergoing total hip arthroplasty. While two primary outcomes were specified prior to enrollment, no specific data analysis plan was defined prospectively. All analyses were intention-to-treat. Continuous, normally distributed data are reported as mean ± standard deviation. Categorical or continuous data not normally distributed are reported as median (10th-90th percentiles) or percent, as appropriate. Comparisons of independent samples were performed using a two-tailed Mann-Whitney U test. The chi-square test and Fisher's exact test were used for differences in proportions, as appropriate. P<0.05 was considered statistically significant for the primary outcomes. Adjustments were not made for multiple comparisons. The results of comparisons in secondary outcomes must be interpreted as suggestive, requiring confirmation in a future trial before considering them as definitive. Prism 10.4.1 (GraphPad, Boston, Massachusetts, USA) was used for all analyses.

## Results

**Figure 2:**
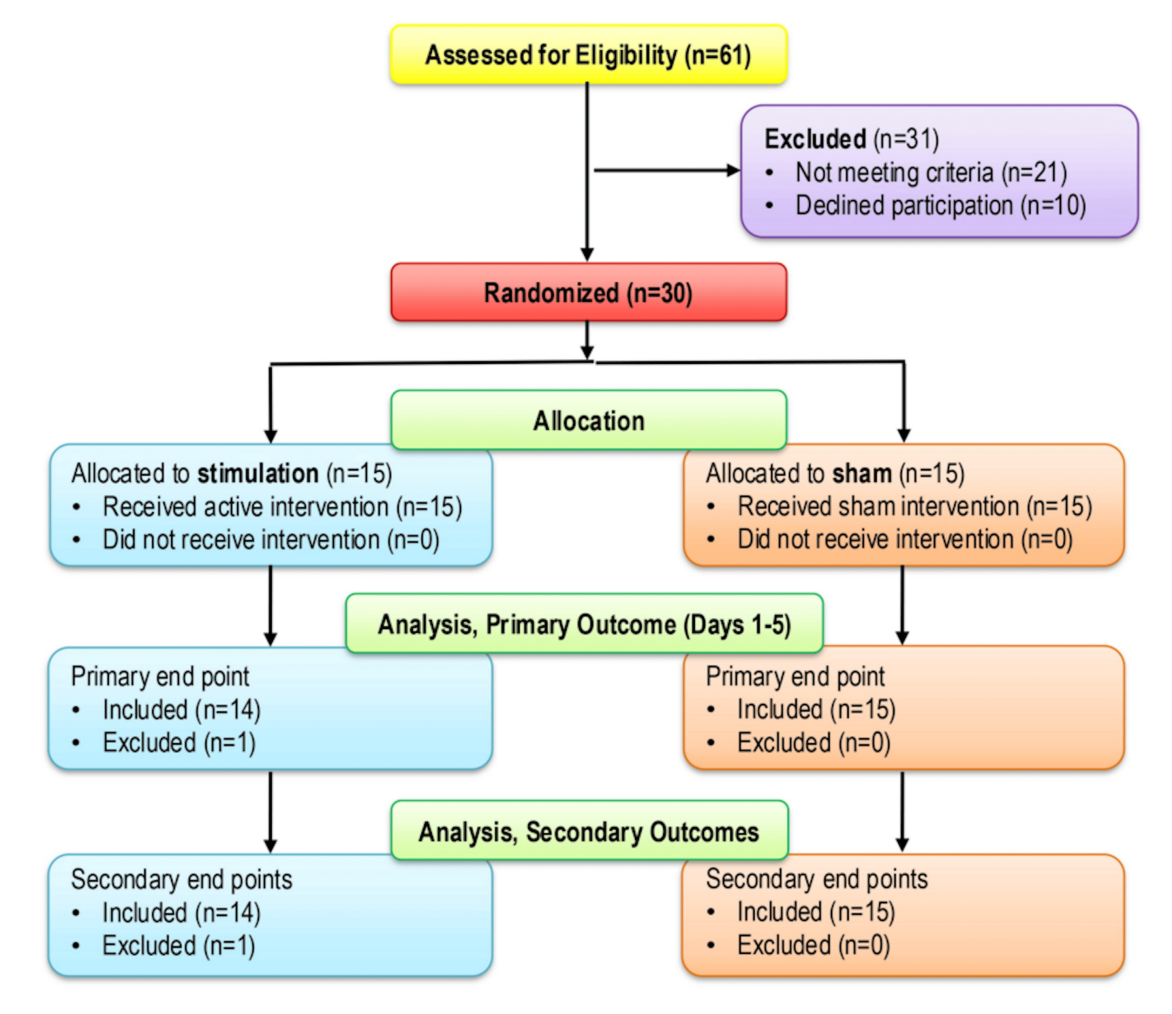
Consolidated Standards of Reporting Trials (CONSORT) diagram Adapted from Schulz et al. [15]

Between October 2022 and July 2023, a total of 30 participants were enrolled (Table 1), randomized to either active stimulation (n=15) or sham (n=15), and had a neuromodulation device applied successfully (Figure 2). One participant randomized to active treatment removed the device the morning of postoperative day one and withdrew from the study prior to any data collection. The remaining 29 participants were included in the analysis.

**Table 1:** Population, procedural information, and day of discharge

Variables	Active (n=15)	Sham (Placebo) (n=15)
Age (years)	65 (7)	72 (6)
Female (%)	33% (5)	53% (8)
Height (cm)	172 (10)	168 (9)
Weight (kg)	80 (17)	73 (13)
Body mass index (kg/m^2^)	27 (4)	26 (4)
Surgical laterality: left hip	43% (7)	27% (4)
Device laterality: left ear	33% (5)	33% (5)
Device and surgical knee same side	73% (11)	93% (14)
Device electrode repositioned	7% (1)	7% (1)
Surgery duration (minutes)	81 (25)	80 (14)
Day of discharge		
0	93% (14)	73% (11)
1	7% (1)	20% (3)
2–3	0% (0)	7% (1)

For the first primary outcome measure, the median (IQR) pain level in the first five days for those receiving active stimulation (n=14) was 2.5 (1.0, 3.8) versus 3.0 (1.9, 4.0) for the sham group (n=15) (P=0.721). Concurrently, the median oxycodone use for the active stimulation group was 3.5 mg (0.1, 9.5) compared to 9.0 mg (2.0, 15.3) for the sham group (P=0.263).

**Figure 3:**
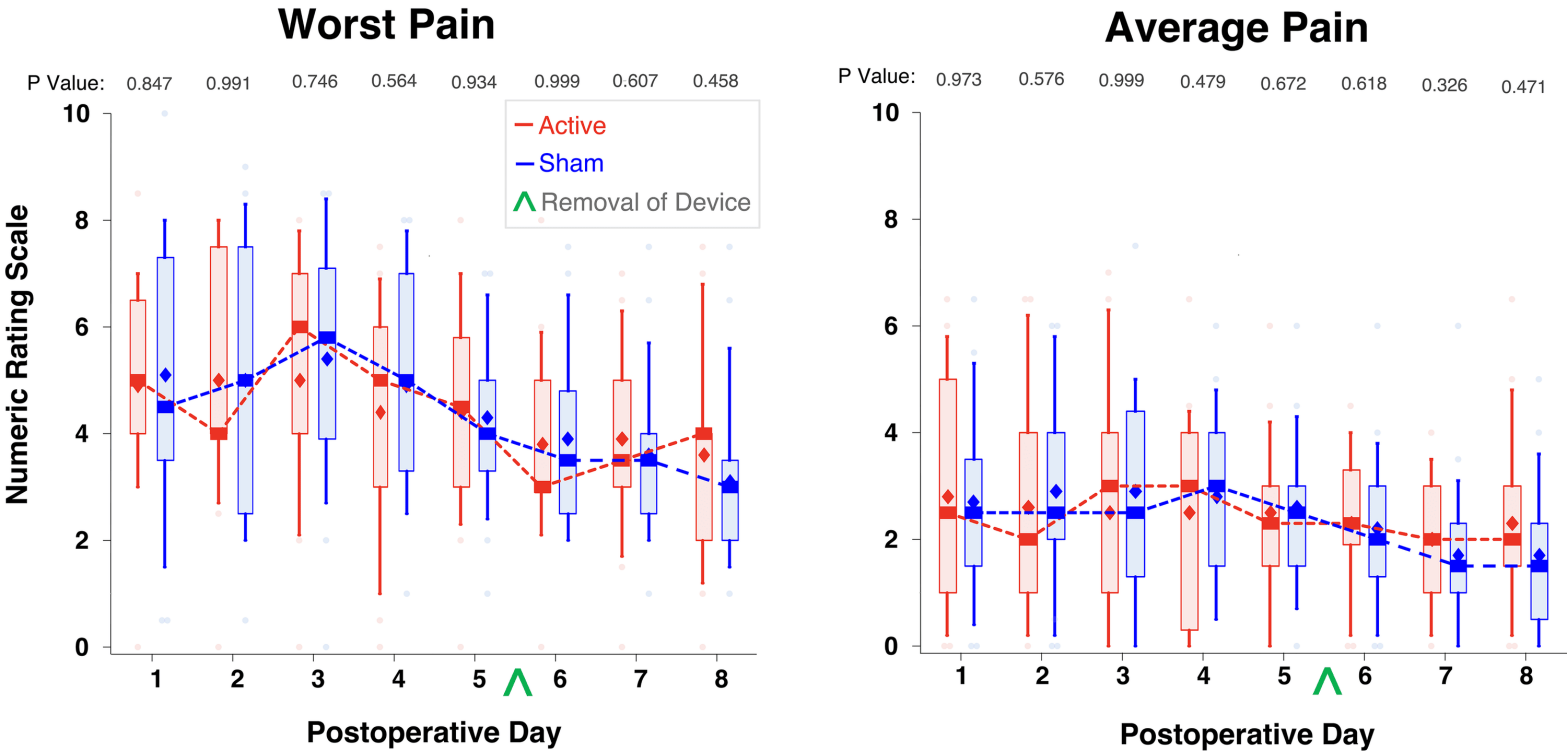
The effects of a five-day percutaneous auricular nerve stimulation on the daily average and worst pain levels Pain severity was measured using a numeric rating scale, with 0 = no pain and 10 = the worst imaginable pain. Data were expressed as medians (dark horizontal bars) with 25th-75th percentiles (box), 10th-90th percentiles (whiskers), mean (diamonds), and outliers (circles). The asterisks denote P<0.05.

**Figure 4:**
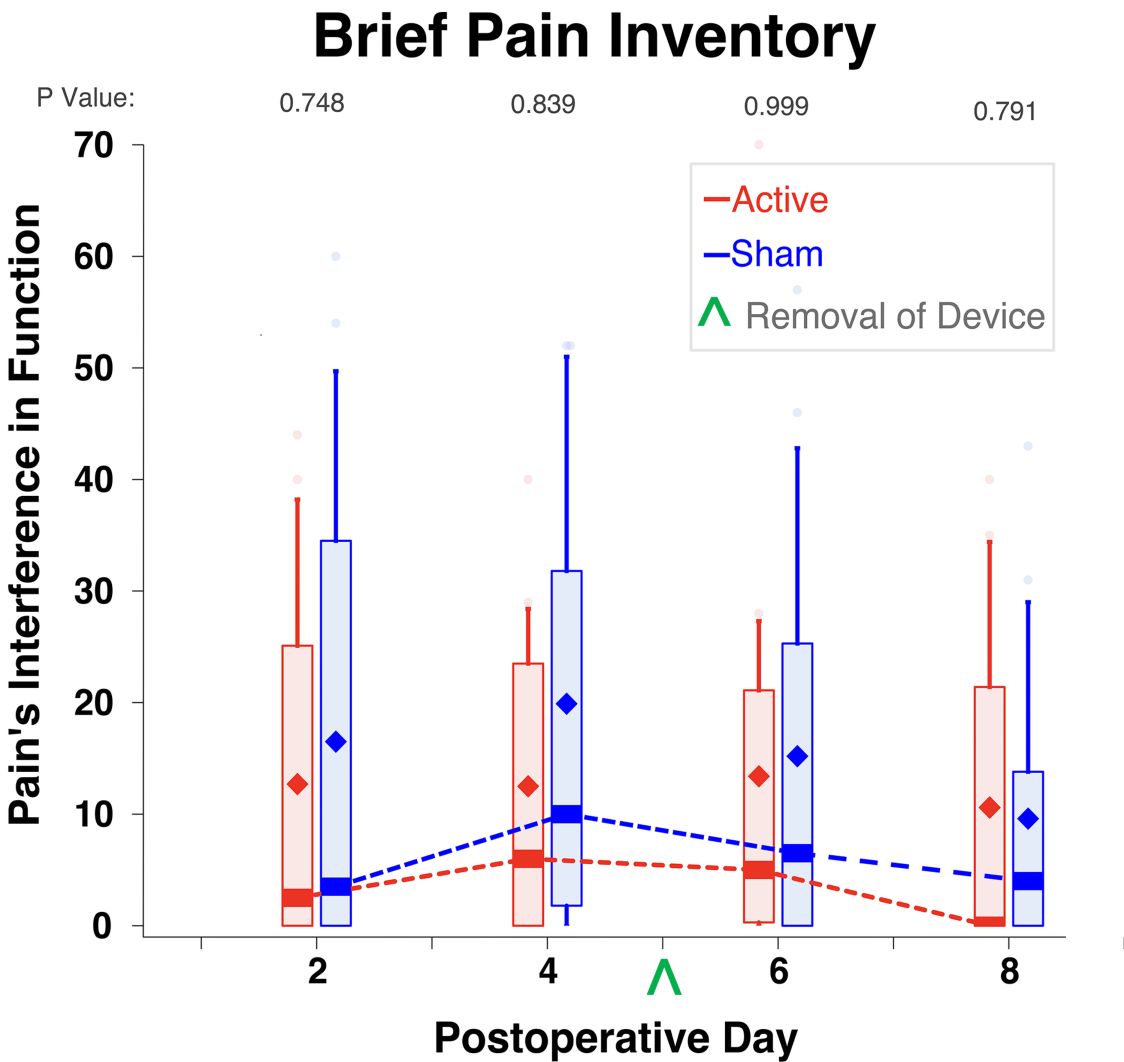
The effects of a five-day percutaneous auricular nerve stimulation on the Brief Pain Inventory (interference domain) Pain interference was indicated using a numeric rating scale of 0-70, with 0 = no pain and 70 = maximal interference. Data were expressed as medians (dark horizontal bars) with 25th-75th percentiles (box), 10th-90th percentiles (whiskers), mean (diamonds), and outliers (circles). The asterisks denote P<0.05.

The differences between the two treatment groups did not reach statistical significance for any of the secondary outcome measures, including daily average and worst (maximum) pain (Figure 3) and physical and emotional interference due to pain as measured using the Brief Pain Inventory (Figure 4). Similarly, the mean (SD) of awakenings due to pain across all eight postoperative nights for participants given active stimulation was 3.4 (3.6) versus 5.9 (12.9) for those given sham (P=0.880).

A total of six participants (20%), three in each treatment group, removed their devices early due to either discomfort while sleeping or specific leads. All participants continued with data collection and were analyzed within the active treatment group per the intention-to-treat principle. No other adverse events or device-related systemic side effects were identified.

## Discussion

This randomized, double-masked, sham-controlled pilot study failed to provide evidence that percutaneous auricular nerve stimulation improves analgesia, reduces opioid requirements, or diminishes pain's interference with physical and emotional functioning during the first week following hip arthroplasty. These are disappointing findings considering the difficulty and lack of effective options for treating pain following hip arthroplasty and the reports of percutaneous auricular neuromodulation successfully providing analgesia after total knee arthroplasty, hernia repair, and cholecystectomy [5,6].

We have a few possible explanations to offer for the differing findings. It is not simply a difference between orthopedic and soft tissue procedures since auricular neuromodulation successfully treated pain after knee arthroplasty [6]. Similarly, other randomized studies demonstrated differing results for soft tissue procedures, with positive findings for hernia repair and cholecystectomy [5]. However, primarily negative results were observed for colorectal surgery and cesarean section [7,8]. Although we can only speculate, the failure of the current study to identify analgesic improvements following hip arthroplasty may involve the specific innervation of the hip joint or pain intensity following this procedure.

Additionally, the marginal lower opioid consumption and Brief Pain Inventory scores of the active treatment group in the present study may have failed to reach statistical significance due to the limited power of this pilot study. However, even if confirmed with a subsequent definitive trial, it is questionable whether these minor improvements would warrant the time, expense, and frequent discomfort (20% removal rate) of the auricular devices. Finally, our negative findings may be due to inadequate electric field characteristics since the field determines the physiologic effect and is influenced by parameters such as amplitude, pulse duration, frequency, number of electrodes, duty cycle, and anatomic electrode location. This built-in variability greatly constrains the extent to which results from a single clinical trial can be applied to other devices, possibly explaining the diverse outcomes seen across studies [8,16-18]. An example is illustrated by the surgical procedure of cesarean section, in which one study was negative while another was positive, each using a different stimulator with varying characteristics [7,19]. The pulse generator used in this study comes with a built-in 3-volt battery and is compatible with load impedances between 1k and 10k Ω, offering a peak output of 3.2 volts. It functions on a biphasic, symmetrical stimulation cycle at a 0.125 Hz frequency, punctuated by occasional non-stimulating periods of rest.

A key goal of this preliminary study was to lay the groundwork for a larger, definitive clinical trial. To achieve this, three potential modifications to the product may enhance the effectiveness of the treatment. First, allowing patients to self-adjust the pulse generator parameters, such as amplitude, frequency, duty cycle, and pulse duration, improves outcomes. Currently, these parameters are fixed. The benefit of enabling patients to titrate stimulation based on their evolving pain management needs and tolerance to electrical current has been demonstrated in both vagus nerve stimulation [20] and ultrasound-guided percutaneous peripheral nerve stimulation [21]. Second, patients have reported discomfort, or even inability, to sleep on the side with the device due to the rigid, angular design positioned behind the ear. In contrast, a different auricular neuromodulation device with a slimmer, more rounded design has not been associated with this issue [17,18,22-25].

The main limitations of our study are the small sample size and the lack of a pre-defined data analysis plan. However, the uniformly negative results for both primary analgesic outcomes and all secondary endpoints reduce the likelihood of a false negative (Type II error). Nevertheless, these findings require validation in a larger, adequately powered, definitive clinical trial. Additionally, we were unable to confirm whether the electrodes remained properly positioned or whether the devices functioned continuously throughout the entire five-day treatment period, as the current version of the neuromodulation device does not have an indicator light or other means to verify ongoing electrical activity. Incorporating a light-emitting diode (LED) could provide real-time confirmation of device operation.

## Conclusions

In conclusion, while this randomized, controlled pilot study demonstrated the feasibility of using percutaneous auricular nerve stimulation following total hip arthroplasty for both the inpatient and outpatient portions of the postoperative period, it failed to identify improvements in analgesia, opioid-sparing, or pain interference in psychological and physical functioning. These are disappointing findings, given the difficulty of treating post-hip arthroplasty pain and the relatively few available analgesic modalities. Our negative findings may be due to the inadequate power of our sample size or the specific characteristics of the neuromodulation device we used. However, pilot studies with identical sample sizes and using the same stimulation device identified benefits for patients following total knee arthroplasty, hernia repair, and cholecystectomy. Therefore, it remains unclear whether a definitive clinical trial is warranted to investigate its use following total hip arthroplasty. Further research is advisable, possibly with a different auricular neuromodulation device and larger sample sizes.
